# Insulin level, lipid profile, and HOMA index in lean and obese patients with polycystic ovary syndrome

**DOI:** 10.25122/jml-2023-0040

**Published:** 2023-08

**Authors:** Shamim Riadh Mohammed Hussein, Alaa Mohammed Sadiq, Shadan Ali Johar, Alaa Jumaah Manji Nasrawi

**Affiliations:** 1 Department of Obstetrics and Gynecology, Faculty of Medicine, Jaber ibn Hayan Medical University, Najaf, Iraq; 2 Department of Obstetrics and Gynecology, Faculty of Medicine, University Of Kufa, Najaf, Iraq; 3 Department of Obstetrics and Gynecology, Al Najaf Health Directorate, Faculty of Medicine, University of Kufa, Najaf, Iraq; 4 Department of Pediatrics, Faculty of Medicine, University of Kufa, Najaf, Iraq

**Keywords:** polycystic ovarian syndrome, PCOS, lean, obese

## Abstract

Polycystic ovary syndrome (PCOS) is a prevalent endocrinopathy characterized by insulin resistance, hyperinsulinemia, and increased ovarian androgen production. While obesity has been linked to the pathogenesis of PCOS, the condition is also observed in individuals with normal BMI. This cross-sectional study aimed to evaluate the biochemical profile of lean and obese patients with PCOS. Fifty female patients with previously diagnosed PCOS were included in the study, attending the outpatient clinic at AL-Zahraa Teaching Hospital in Al-Najaf between September 2021 and March 2022. Blood samples were collected from each patient to assess insulin levels, lipid profiles, and fasting blood sugar. The results showed a comparable percentage of lean and obese PCOS patients, with a slightly higher proportion of obese individuals. Statistically significant differences were observed in obese patients with higher fasting blood sugar levels, insulin, HDL cholesterol, and triglycerides. Additionally, the HOMA index, an indicator of insulin resistance, was higher in obese individuals. Lean PCOS patients exhibited metabolic, hormonal, and hematopoietic dysregulations comparable to or less pronounced than those affecting the obese phenotype. Regardless of BMI, insulin resistance is part of PCOS and must be treated immediately.

## INTRODUCTION

Polycystic Ovary Syndrome (PCOS) is a prevalent condition that affects the functioning of a woman's ovaries, leading to various symptoms such as infertility, enlarged ovaries, menstrual irregularities, elevated male hormone levels, excess facial and body hair, acne, and obesity [1, 2]. PCOS is characterized by a combination of signs and symptoms, including polycystic ovaries (enlarged ovaries containing many fluid-filled sacs), irregular or absent menstrual periods, elevated levels of male hormones (androgens), and signs of insulin resistance [3]. The exact cause of PCOS remains unknown, but it is believed to involve a complex interplay of genetic and environmental factors, including diet and lifestyle [1, 3].

Insulin resistance (IR) is a key feature observed in many PCOS women, and its severity tends to increase with a higher body mass index (BMI) [4]. The use of insulin sensitizers has shown significant improvement in ovulatory function, menstrual cyclicity, fertility, and various metabolic and endocrine aspects of PCOS [4]. Furthermore, women with PCOS have a higher risk of developing gestational diabetes [5].

Compensatory hyperinsulinemia has multiple effects on the adrenal gland as well as ovaries, promoting androgen production under the stimulation of luteinizing hormone [LH] [6].

The euglycemic hyperinsulinemic clamp technique, a slow procedure for conventional clinical practitioners, is recognized as the gold standard in testing insulin resistance. The Matthews-developed homeostasis model assessment-estimated insulin resistance (HOMA-IR) is utilized in studies to evaluate insulin resistance. HOMA-IR is more practical than the "gold" standard, the euglycemic clamp, for assessing insulin resistance [7]. It is calculated by multiplying fasting plasma insulin (FPI) and fasting plasma glucose (FPG) and then dividing the result by the constant 22.5:

HOMA-IR = (FPI×FPG)/22.5

Insulin sensitivity is indicated by a HOMA-IR result of less than 1.0, suggesting good responsiveness of the body's cells to insulin. A HOMA-IR result between 1.0 and 1.9 signifies early insulin resistance, indicating that the body's cells are starting to show some resistance to the effects of insulin. A HOMA-IR result above 2.9 suggests advanced insulin resistance, indicating severe resistance to insulin's actions and a higher likelihood of developing metabolic disorders such as type 2 diabetes.

Studies have shown that obese women with polycystic ovary syndrome (PCOS) are more prone to dyslipidemia, particularly elevated triglycerides (TG) and decreased high-density lipoprotein cholesterol (HDL-C) [8]. Anthropometric characteristics, such as body mass index (BMI) and hip circumference, are important parameters correlated to lipid profiles in overweight or obese PCOS patients [8]. Lean mass percentages have been found to predict a better metabolic profile in PCOS patients [9].

Both obese and lean women with PCOS have been found to exhibit poorer metabolic health and greater visceral adiposity compared to the control group [10]. Lipid ratios and obesity indices have been identified as effective predictors of metabolic syndrome in PCOS patients [11]. These findings suggest that obesity is an important factor in the development of dyslipidemia in PCOS patients. The aim of this study was to examine the biochemical profile of lean and obese PCOS patients.

## MATERIALS AND METHODS

### Study design and participants

This cross-sectional study included 50 female patients with previously diagnosed PCOS who visited the outpatient clinic at AL-Zahraa teaching hospital in Al-Najaf from September 2021 to March 2022. The inclusion criteria for this study included any female patient who had previously been clinically diagnosed with PCOS and expressed willingness to participate. Exclusion criteria comprised diabetic patients and those currently taking hormonal treatment or metformin or who had taken these medications within the last 4 months.

### Methods

Venous blood samples of 5 ml were collected from each participant while they were in a sitting position, following a fasting period of at least 8 hours. The blood samples were used to measure several parameters, including fasting serum glucose, lipid profile (cholesterol, triglyceride, HDL, LDL, and VLDL), and insulin levels. The insulin levels were assessed using the Enzyme-Linked Immune Sorbent Assay (ELISA) method with the Rosh e411 analyzer. Additionally, anthropometric data for each participant, including waist circumference using a tape measure and weight using an electronic scale, were recorded.

### Calculation of Body Mass Index (BMI)

Body mass index (BMI) was calculated by dividing the weight in kilograms by the square of the height in meters:

BMI = weight (kg)/height (m^2^) [12]

BMI subgroups were categorized as follows: BMI≤24.9 kg/m^2^ (normal weight), BMI ranging from 25 to 29.9 kg/m^2^ (overweight), and BMI more than 30 kg/m^2^ (obese) [13].

### Homeostasis model assessment of insulin resistance (HOMA-IR)

HOMA-IR was used to evaluate insulin resistance using the equation below:

HOMA-IR=(Fasting serum insulin (µU/ml) × fasting plasma glucose (mg/dl)/22.5)

### Statistical analysis

Data were analyzed using SPSS version 25. Descriptive statistics were used to summarize the data, including frequency, percentage, mean, and standard deviation. The independent t-test was used to assess the mean level differences between the two groups. Histogram figures were employed to compare the means of the two groups. The Pearson correlation test (r) was used to evaluate the correlation between numerical data, with values <0.2 indicating weak correlation, values between 0.2 and 0.8 showing moderate correlation, and values >0.8 indicating strong correlation. To visually represent the correlation, scatter dot diagrams were used. Additionally, other variables between the two groups were analyzed using the independent t-test, with a p-value of <0.05 considered statistically significant.

## RESULTS

**Figure 1:**
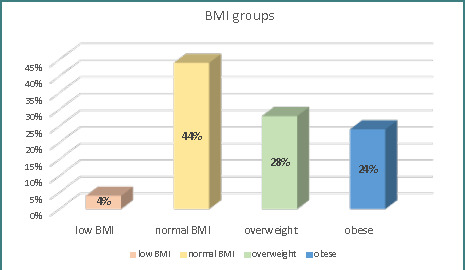
BMI grops of patients

The study included 50 female patients with PCOS, with a mean BMI of 27.50±7.68, ranging from 17.33 to 50 kg/m^2^. The patients were categorized into different BMI groups, as shown in Figure 1.

An independent t-test was performed to compare the mean differences between obese and lean PCOS patients. The results revealed that obese PCOS patients had statistically higher mean levels of FBS (105.76±17.93 mg/dl), triglycerides (86.96±20.26 mg/dl), and HOMA-IR (6.44±5.67) compared to lean PCOS patients (97.50±16.49, 64.50±17.40, 22.17±1.93, 3.74±1.35) with p-values <0.0001, <0.0001, and 0.02, respectively. On the other hand, lean PCOS patients had a statistically significantly higher mean level of HDL (51.65±7.73) compared to obese PCOS patients (45.03±8.55) with a p-value of 0.006, as presented in Table 1.

**Table 1:** Mean difference of FBS, triglyceride, cholesterol, HDL, VLDL, LDL, BMI, and IR between lean and obese patients

	groups	N	Mean ± SD	SE	p-value
FBS (mg/dl)	lean PCOS	24	97.50±16.49	3.36	0.05*
	obese PCOS	26	105.76±17.93	3.51	
insulin (µIU/ml)	lean PCOS	24	15.96±6.53	1.33	0.06
	obese PCOS	26	23.74±19.29	3.78	
Triglyceride (mg/dl)	lean PCOS	24	64.50±17.40	3.55	<0.0001*
	obese PCOS	26	86.96±20.26	3.97	
cholesterol (mg/dl)	lean PCOS	24	143.25±30.17	6.15	0.53
	obese PCOS	26	147.90±17.27	3.38	
HDL (mg/dl)	lean PCOS	24	51.65±7.73	1.57	0.006*
	obese PCOS	26	45.03±8.55	1.67	
VLDL (mg/dl)	lean PCOS	24	17.65±7.47	1.52	0.28
	obese PCOS	26	19.96±7.70	1.51	
LDL (mg/dl	lean PCOS	24	91.13±22.29	4.55	0.99
	obese PCOS	26	91.06±17.46	3.42	
BMI (kg/m^2^)	lean PCOS	24	22.17±1.93	0.39	<0.0001*
	obese PCOS	26	32.42±7.72	1.51	
HOMA-IR	lean PCOS	24	3.74±1.35	0.27	0.02*
	obese PCOS	26	6.44±5.67	1.11	

* p-value≤0.05

Further analysis based on the HOMA-IR index revealed that 6 patients were insulin-sensitive, while 44 were insulin-resistant. Patients with insulin resistance had a statistically significant higher mean of FBS and insulin (104.61±16.37, 21.37±15.51) compared to insulin-sensitive patients (79.16±8.20, 7.67±0.12) with p-values 0.001 and 0.03, respectively, as presented in Table 2..

**Table 2:** Mean difference of FBS, triglyceride, cholesterol, HDL, VLDL, LDL, BMI, and IR between insulin-sensitive and resistant patients

		N	Mean ± SD	SE	p-value
FBS (mg/dl)	insulin sensitive	6	79.16±8.20	3.35	0.001*
	insulin resistance	44	104.61±16.37	2.46	
insulin (µIU/ml)	insulin sensitive	6	7.67±0.12	0.05	0.03*
	insulin resistance	44	21.37±15.51	2.33	
Triglyceride (mg/dl)	insulin sensitive	6	72.60±19.98	8.15	0.67
	insulin resistance	44	76.67±22.33	3.36	
cholesterol (mg/dl)	insulin sensitive	6	134.00±44.81	18.29	0.21
	insulin resistance	44	147.25±20.29	3.05	
HDL (mg/dl)	insulin sensitive	6	51.31±12.43	5.07	0.35
	insulin resistance	44	47.78±8.23	1.24	
VLDL (mg/dl)	insulin sensitive	6	16.40±4.84	1.97	0.40
	insulin resistance	44	19.19±7.88	1.18	
LDL (mg/dl)	insulin sensitive	6	93.80±8.91	3.64	0.72
	insulin resistance	44	90.72±20.80	3.13	
BMI (kg/m^2^)	insulin sensitive	6	22.09±3.75	1.53	0.06
	insulin resistance	44	28.24±7.80	1.17	

* p-value≤0.05

**Figure 2:**
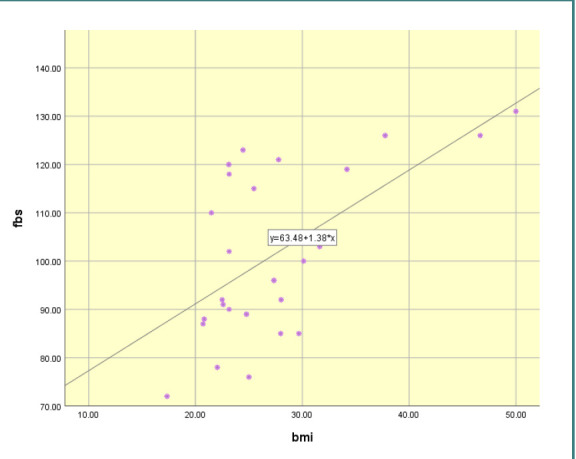
Scattered dot diagram representing the correlation between BMI and FBS

**Figure 3:**
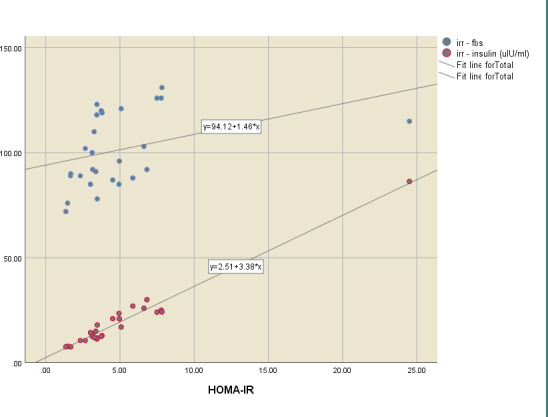
Scattered dot diagram representing the correlation between HOMA-IR with insulin and FBS

Moreover, Pearson correlation analysis evaluated the correlation between BMI, HOMA-IR, and other variables. The results showed a statistically significant positive moderate correlation between BMI and FBS (r=0.59, p<0.001) (Figure 2). HOMA-IR also had a statistically positive weak correlation with FBS (r=0.34, p=0.01) and a strong positive correlation with insulin (r=0.98, p<0.001), as presented in Table 3 and Figure 3.

**Table 3:** Correlation between BMI, HOMA-IR, and other biochemical variables

		BMI	HOMA-IR
FBS (mg/dl)	Pearson Correlation	0.597^**^	0.347^*^
	p-value	<0.001	0.013
insulin (µIU/ml)	Pearson Correlation	0.108	0.983^**^
	p-value	0.456	<0.001
Triglyceride (mg/dl)	Pearson Correlation	0.257	0.230
	p-value	0.071	0.108
cholesterol (mg/dl)	Pearson Correlation	0.238	-0.004
	p-value	0.097	0.978
HDL (mg/dl)	Pearson Correlation	-0.248	-0.003
	p-value	0.083	0.982
VLDL (mg/dl)	Pearson Correlation	0.002	0.050
	p-value	0.989	0.732
LDL (mg/dl)	Pearson Correlation	0.167	-0.126
	p-value	0.246	0.383
BMI (kg/m^2^)	Pearson Correlation	1	0.219
	p-value		0.127
HOMA-IR	Pearson Correlation	0.219	1
	p-value	0.127	

* Correlation is significant at the 0.05 level (2-tailed); **Correlation is significant at the 0.01 level (2-tailed); p-value≤0.05

## DISCUSSION

Even though most PCOS cases are obese or overweight, a small but significant minority of patients have a normal body mass index which makes the diagnosis process and the therapeutic approach more challenging.

In the current study, over half of the participants were obese or overweight, and the remaining patients had normal BMI, consistent with previous research by Ghatnatti V. *et al*. [14]. However, Deng Y *et al*. [15] reported a lower percentage of lean patients (33%).

Obese PCOS patients had a higher mean of FBS that agreed with other studies [15-17] and disagreed with Shirazi FK *et al*. [18], who observed no difference between the groups.

There was no difference in mean insulin level between the groups, consistent with previous studies [19, 20]. However, Jamil AS and colleagues reported higher insulin levels in the fasting state and after meals, suggesting a link between insulin and central androgen actions that may impact progesterone's regulation of the GnRH pulse generator [21].

Regarding lipid profile, the current study revealed higher triglyceride levels and low HDL in obese PCOS patients, with no differences in other lipids parameters. In another study, the difference in lipid profile was observed only in TG, TC, and LDL, with no difference in HDL mean [22]. In a case-control study, the lipid profile of lean and obese patients was significantly higher than the control group with normal and high BMI [23]. A cluster of interconnected pathogenic modalities, including obesity, hyperinsulinemia, oxidative stress, anovulation, and hyperandrogenemia, can cause dyslipidemia in PCOS. These parameters slightly impact differences in lipid profiles among studies since insulin is one of the main regulators of lipoprotein lipase activity, and hyperandrogenemia has an independent function in lipoprotein and lipid metabolism [24].

Both obese and lean patients had abnormally high HOMA-IR; however, obese patients had worse insulin resistance than lean patients. A similar finding was observed by Ghatnatti V *et al*. [14], while Gholinezhad M *et al*. [25] found a statistically significant association between obese PCOS patients and higher HOMA-IR values. The study also observed a significant association between BMI and increased lipid profile. IR and higher BMI are tightly related, but these may also be found in PCOS individuals with normal weight. Hyperandrogenism and persistent anovulation are linked to the development of IR in individuals with PCOS. Increased total and free androgens result from insulin's promotion of ovarian androgen synthesis and reduced hepatic output of sex hormone-binding globulin (SHBG). IR occurs in normal-weight women as well, it is linked to fat distribution and plays a significant role in PCOS among teenagers [26].

In our study, we observed that insulin levels were higher in the insulin resistance group, which aligns with previous research by Saghafi-Asl M *et al*. [27] and Ebrahimi-Mamaghani *et al*. [28]. They also reported that impaired glucose tolerance (IGT) and type 2 diabetes mellitus (T2DM) can occur as a result of insufficient insulin production in response to insulin resistance (IR). Contradictory findings have emerged from investigations on beta-cell function in women with PCOS. Compared to controls of the same age and BMI in a European population, women with PCOS secreted more insulin to maintain healthy glucose homeostasis, which explains the current finding [18].

There was no difference in lipid profile markers in insulin-sensitive or insulin-resistance groups. These findings are consistent with previous research, where no statistically significant differences were observed in lipid profile markers between these groups, except for a higher level of high-density lipoprotein cholesterol (HDL-C) in the insulin-sensitive group [27]. Similarly, another study [28] reported no significant difference in the mean lipid profile except for HDL.

In contrast to our findings, Anuradha K *et al*. [29] reported higher triglycerides, total cholesterol, and lower high-density lipoprotein (HDL) in insulin resistance patients. The difference could be related to the levels of sex hormones, which were not evaluated in the current study.

The small sample size and study design are some of the limitations of the current study. The restricted number of participants may have affected the statistical power and generalizability of our results. Moreover, the lack of hormonal profile evaluation in our investigation is another limitation due to poor cooperation. Finally, it is important to acknowledge that not all populations experience dyslipidemia in the same way. The interplay effects of hyperandrogenism, insulin resistance, environmental variables, and heredity likely lead to various kinds of dyslipidemia. Atherogenic dyslipidemia was a prevalent symptom in research where women with PCOS were included.

## CONCLUSION

Lean PCOS patients have a range of metabolic, hormonal, and hematopoietic dysregulations, although these manifestations are often comparable to or less pronounced than those observed in the obese phenotype. Regardless of BMI, insulin resistance remains a prominent feature of PCOS and should be promptly addressed in all affected individuals.

## Data Availability

Further data is available from the corresponding author upon reasonable request.
